# GABA and GABA-Alanine from the Red Microalgae *Rhodosorus marinus* Exhibit a Significant Neuro-Soothing Activity through Inhibition of Neuro-Inflammation Mediators and Positive Regulation of TRPV1-Related Skin Sensitization

**DOI:** 10.3390/md16030096

**Published:** 2018-03-17

**Authors:** Amandine Scandolera, Jane Hubert, Anne Humeau, Carole Lambert, Audrey De Bizemont, Chris Winkel, Abdelmajid Kaouas, Jean-Hugues Renault, Jean-Marc Nuzillard, Romain Reynaud

**Affiliations:** 1 Active Beauty Department, Givaudan France, 51110 Pomacle, France; anne.humeau@givaudan.com (A.H.); carole.lambert@givaudan.com (C.L.); audrey.de_bizemont@givaudan.com (A.D.B.); romain.reynaud@givaudan.com (R.R.); 2 Institut de Chimie Moléculaire de Reims, UMR CNRS 7312, Université de Reims Champagne-Ardenne, 51687 Reims Cedex 2, France; jh.renault@univ-reims.fr (J.-H.R.); jm.nuzillard@univ-reims.fr (J.-M.N.); 3 Givaudan Nederland, Flavors, 1411 GP Naarden, The Netherlands; chris.winkel@givaudan.com (C.W.); abdelmajid.kaouas@givaudan.com (A.K.)

**Keywords:** microalgae, *Rhodosorus marinus*, neuro-inflammation, pro-inflammatory cytokines, TRPV1 receptor, pain sensing, NMR-based dereplication

## Abstract

The aim of the present study was to investigate the neuro-soothing activity of a water-soluble hydrolysate obtained from the red microalgae *Rhodosorus marinus* Geitler (Stylonemataceae). Transcriptomic analysis performed on ≈100 genes related to skin biological functions firstly revealed that the crude *Rhodosorus marinus* extract was able to significantly negatively modulate specific genes involved in pro-inflammation (interleukin 1α encoding gene, IL1A) and pain detection related to tissue inflammation (nerve growth factor NGF and its receptor NGFR). An in vitro model of normal human keratinocytes was then used to evaluate the ability of the *Rhodosorus marinus* extract to control the release of neuro-inflammation mediators under phorbol myristate acetate (PMA)-induced inflammatory conditions. The extract incorporated at 1% and 3% significantly inhibited the release of IL-1α and NGF secretion. These results were confirmed in a co-culture system of reconstructed human epithelium and normal human epidermal keratinocytes on which a cream formulated with the *Rhodosorus marinus* extract at 1% and 3% was topically applied after systemic induction of neuro-inflammation. Finally, an in vitro model of normal human astrocytes was developed for the evaluation of transient receptor potential vanilloid 1 (TRPV1) receptor modulation, mimicking pain sensing related to neuro-inflammation as observed in sensitive skins. Treatment with the *Rhodosorus marinus* extract at 1% and 3% significantly decreased PMA-mediated TRPV1 over-expression. In parallel with these biological experiments, the crude *Rhodosorus marinus* extract was fractionated by centrifugal partition chromatography (CPC) and chemically profiled by a recently developed ^13^C NMR-based dereplication method. The CPC-generated fractions as well as pure metabolites were tested again in vitro in an attempt to identify the biologically active constituents involved in the neuro-soothing activity of the *Rhodosorus marinus* extract. Two active molecules, namely, γ-aminobutyric acid (GABA) and its structural derivative GABA-alanine, demonstrated a strong capacity to positively regulate skin sensitization mechanisms related to the TRPV1 receptors under PMA-induced inflammatory conditions, therefore providing interesting perspectives for the treatment of sensitive skins, atopia, dermatitis, or psoriasis.

## 1. Introduction

Skin is the first defensive barrier to protect the body against external aggressions including, for instance, temperature variation, bacterial/viral infections, or exposure to chemicals. Cutaneous stress can result in disruption of skin homeostasis and the appearance of severe disorders at different cellular and molecular levels. The epidermis, which is composed of very cohesive keratinocytes organized in various differentiation degrees from the basal layer to the corneocytes, plays a significant role as a physical barrier [1,2]. The epidermis is also a metabolically active tissue involved in the regulation of lipid biosynthesis which promotes skin impermeability and barrier formation [3,4].

Some types of skin are subjected to barrier function impairments that promote cutaneous permeability [5]. In this case, the skin responds with an inflammatory reaction led by keratinocytes, skin immune cells, and recruitment of macrophages and neutrophils for the release of pro-inflammatory cytokines such as IL-1α, IL-1B, IL-6, IL-8, and CCL5 [6]. This chronic inflammation process results in roughness, dryness, burning, and redness that represent the physical features of sensitive and atopic skins [7]. Sensitive skins are also generally predisposed to hyperalgesia promoted by the secretion of neuro-active molecules such as the nerve growth factor (NGF), substance P, or calcitonin gene-related peptide (CGRP) [8]. NGF is a molecule secreted by keratinocytes [9] and epidermal nerve endings that participate in pain sensing [10,11,12]. Increased production of NGF up-regulates TRPV1 (transient receptor potential vanilloid 1) expression in epidermal nerve endings, leading to hypersensitization [13,14]. Many people are suffering from sensitive skin. Consequently, intense research efforts are currently being made in the pharmaceutical and dermo-cosmetic industries in order to better understand the molecular and cellular mechanisms involved in sensitive-skin-associated disorders and to find new efficient treatments. 

In the present work, a specific in vitro model was developed for the evaluation of TRPV1 receptor modulation in response to neuro-inflammation induced by a pro-inflammatory stimulus performed on normal human astrocytes (NHA). Using this model, the neuro-soothing activity of a water-soluble hydrolysate obtained from *Rhodosorus marinus* was investigated. *Rhodosorus marinus* Geitler (Stylonemataceae) is a unicellular red microalga belonging to the phylum Rhodophyta, and commonly found in tropical and subtropical seas. To date, *Rhodosorus marinus* has been mainly exploited as a source of phycobiliproteins, in particular, phycoerythrin, phycocyanin, and allophycocyanin, which are water-soluble light-harvesting pigments [15,16,17]. A study conducted in 2001 revealed an inhibitory effect of the ethanol-insoluble fraction of *Rhodosorus marinus* on hyaluronidase activation, suggesting a potential effect in anti-inflammatory and anti-allergic reactions [18]. Our objective was therefore to establish the chemical profile of the most polar *Rhodosorus marinus* metabolites and evaluate their inhibitory activity against inflammation and neuro-inflammation processes. 

In a first step, a water-soluble hydrolysate was produced from a photo-bioreactor culture of *Rhodosorus marinus*. The crude extract was analyzed by transcriptomics on Normal Human Epidermal Keratinocytes to examine the expression profiles of ≈100 genes related to skin biological functions. Based on transcriptomic data, the inhibitory effect of the *Rhodosorus marinus* extract was investigated on TRPV1 receptor over-expression in response to neuro-inflammation. In parallel, the crude extract was fractionated by centrifugal partition chromatography (CPC) and the metabolite profile of the simplified mixtures obtained was established by a recently developed ^13^C NMR-based dereplication method. The CPC-generated fractions as well as pure metabolites were tested again in vitro in an attempt to identify the biologically active constituents involved in the neuro-soothing activity of the *Rhodosorus marinus* extract. 

## 2. Results and Discussion

**Figure 1:**
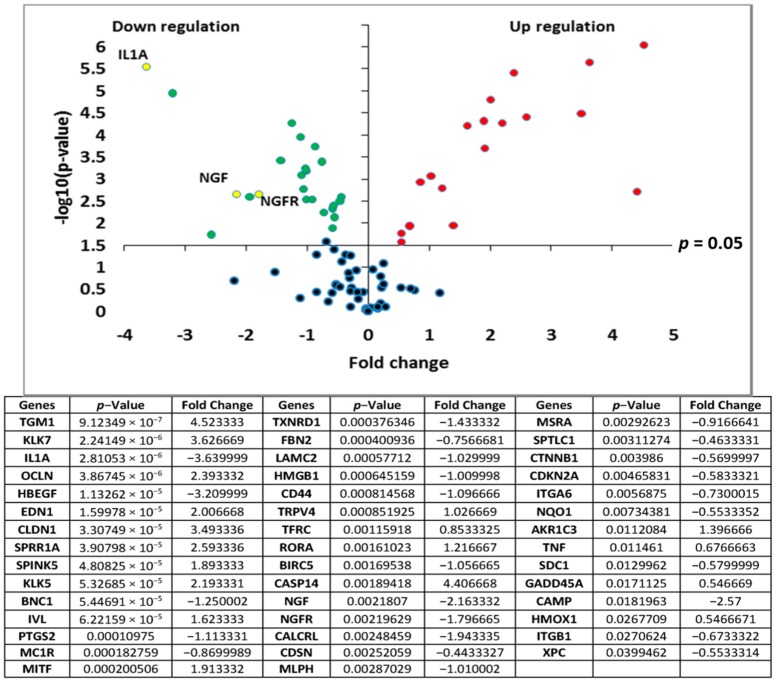
Transcriptomic analysis of the *Rhodosorus marinus* extract (RDMS) using a TaqMan card targeting epidermis functions.

Transcriptomic analyses of the polar *Rhodosorus marinus* extract were performed on normal human epidermal keratinocytes using the TaqMan card which targets epidermis functions, to determine if the whole extract at 3% could have potential activity against neuro-inflammation. As illustrated in Figure 1, a total of 44 genes were significantly modulated by the *Rhodosorus marinus* extract after 24 h of treatment. Among them, IL1A, NGF, and NGFR were down-regulated by a factor of −3.6 (*p* < 0.001), −2.2 (*p* < 0.01), and −1.8 (*p* < 0.01), respectively (Figure 1). The IL1A gene encodes the pro-inflammatory cytokines IL-1α, while NGF and NGFR encode proteins involved in the survival and differentiation of nerve cells, especially sensory neurons involved in pain detection related to tissue inflammation [19,20]. 

The results of this gene expression analysis therefore suggest that the *Rhodosorus marinus* extract could efficiently control the neurogenic inflammation associated with skin disorders such as psoriasis, dermatitis, and atopia.

**Figure 2:**
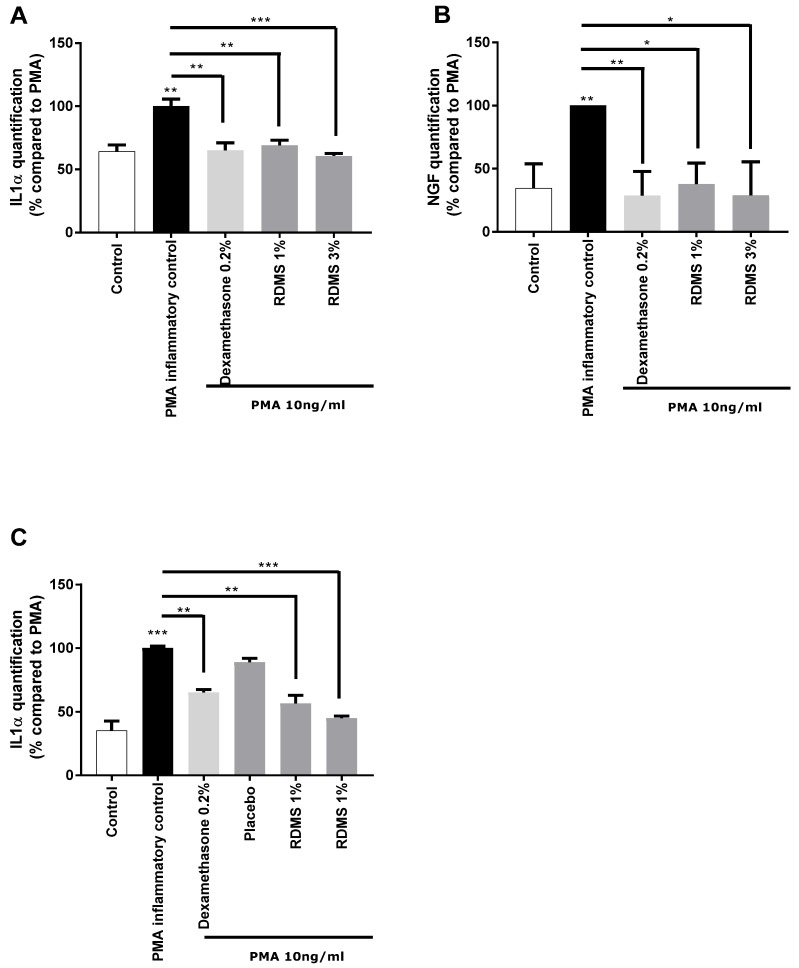
Impact of the *Rhodosorus marinus* (RDMS) extract on neuro-inflammation on normal human epidermal keratinocytes **(**NHEK) and reconstructed human epithelium (RHE). (**A**) IL-1α quantification after RDMS treatment on NHEK. (**B**) Nerve growth factor (NGF) quantification after RDMS treatment on NHEK. (**C**) IL-1α quantification after topical RDMS treatment on RHE. Statistical analyses were performed using Student’s *t*-test with *p* < 0.05 *, *p* < 0.01 ** and *p* < 0.001 ***. PMA: Phorbol Myristate Acetate.

In a second step, a more specific model of neuro-inflammation was used to evaluate the ability of the *Rhodosorus marinus* extract to modulate the release of IL-1α and NGF in normal human keratinocytes. The cells were cultured in inflammatory conditions mediated by phorbol myristate acetate (PMA), a chemical agent commonly used in similar biological models as an activator of inflammation. As shown in Figure 2, PMA treatment induced a significant release of IL-1α and NGF molecules that mimic neuro-inflammation in vitro. Treatment with the anti-inflammatory reference dexamethasone significantly reduced the PMA-induced neuro-inflammation as observed from the decrease of IL1α and NGF release in the culture medium (Figure 2A,B). Besides this, the *Rhodosorus marinus* extract incorporated at 1% and 3% induced a significant decrease of the IL-1α and NGF releases, indicating that some metabolites present in the extract could limit the neuro-inflammation process in this in vitro model. 

In a third step, an original ex vivo model based on a co-culture system of reconstructed human epithelium (RHE) and normal human epidermal keratinocytes **(**NHEK) was developed (Figure 2C). This model enables the topical application of a formulated product on RHE to induce neuro-inflammation by PMA in the bottom compartment containing NHEK, and to evaluate the ability of the product to penetrate the skin and thus protect the NHEK against neuro-inflammation. The results showed that PMA induced a significant increase in IL-1α release. This inflammatory process was significantly reduced by the anti-inflammatory reference, dexamethasone, proving the induction of inflammation is this model and thus validating the experiment (Figure 2C). A topical application of two creams formulated with the *Rhodosorus marinus* extract at 1% and 3% significantly reduced the inflammation as revealed by the inhibition of IL-1α release in the bottom compartment containing NHEK, while the cream placebo did not exert any effect. These data demonstrate that the observed anti-inflammatory effect undoubtedly resulted from the bioactivity of the *Rhodosorus marinus* extract, and that some metabolites of the extract were able to protect against inflammation. 

As sensitive skin is also associated with pain sensation and discomfort, the fourth step of this work was focused on the specific TRPV1 receptor (transient receptor potential cation channel subfamily V member 1) which is over-expressed in the case of pain sensing. The detection of pain is in fact induced by the activation of TRPV1 expressed in the skin nerve endings, leading to a calcium influx transmitted as a pain signal via the neuron membrane to the brain [21,22,23]. 

**Figure 3:**
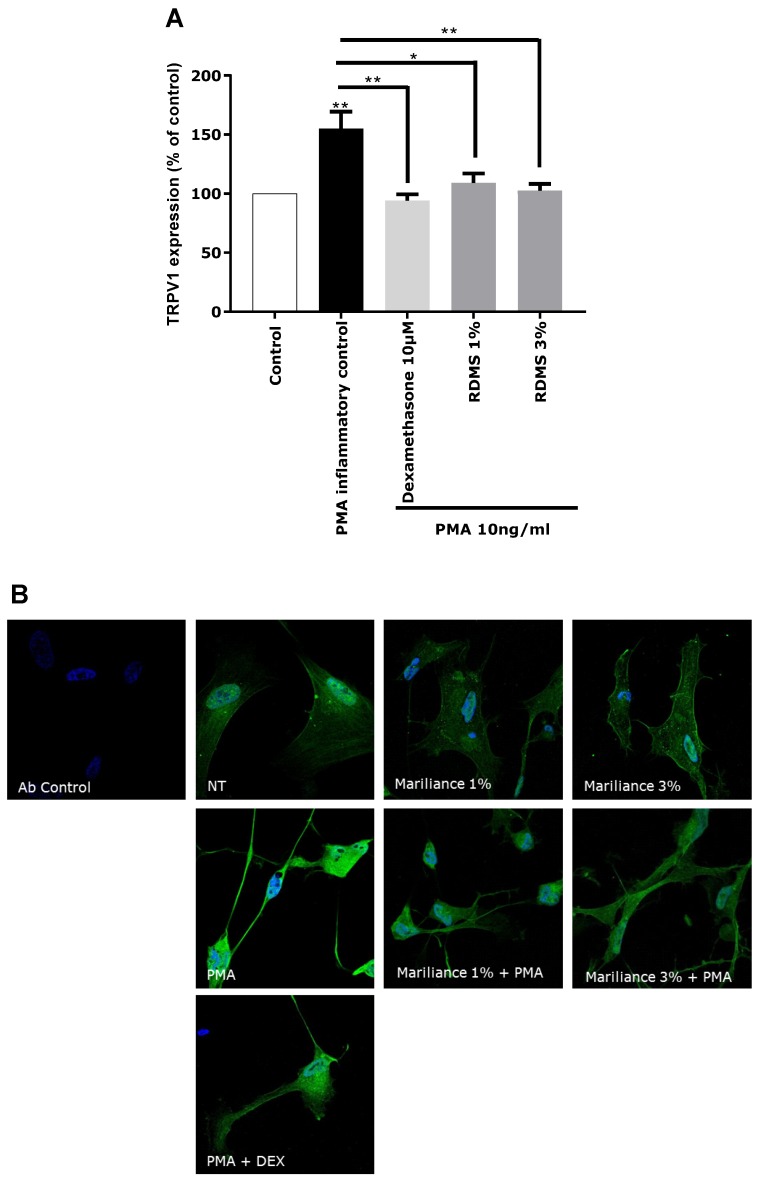
Impact of the *Rhodosorus marinus* extract on Transient Receptor Potential Vanilloid 1 (TRPV1) expression in normal human astrocytes (NHA). (**A**) TRPV1 quantification by specific immunostaining of TRPV1 after treatment with RDMS at 1% and 3%. Statistical analyses were performed using Student’s *t*-test with *p* < 0.05 *, *p* < 0.01 **. (**B**) Confocal microscopy visualization of RDMS impact on TRPV1 over-expression induced by PMA.

In our in vitro model, TRPV1 over-expression was mediated by an inflammatory stimulus in order to correlate the TRPV1 activation to inflammation and to mimic as much as possible a pain sensation related to neuro-inflammation as observed in sensitive skin. Normal human astrocytes (NHA) were used as a neuron-like cell model. These human cells have the particularity of expressing the TRPV1 receptor and participating in the neuronal system. An over-expression of TRPV1 was induced in NHA by PMA-mediated inflammation. This TRPV1 activation in NHA under inflammatory conditions confirmed that our in vitro model is relevant in reflecting pain sensing related to neuro-inflammation. As observed in Figure 3, the over-expression of TRPV1 was significantly reduced by the anti-inflammatory reference, dexamethasone. Treatment with the *Rhodosorus marinus* extract at 1% and 3% significantly decreased the PMA-mediated TRPV1 over-expression, but with no significant difference between the two concentrations. These results demonstrated that the *Rhodosorus marinus* extract can control pain sensing related to neuro-inflammation in vitro through a decrease of TRPV1 expression.

In an attempt to identify the *Rhodosorus marinus* metabolites involved in the observed neuro-inflammation inhibitory activity, the crude extract was fractionated by centrifugal partition chromatography (CPC) and the obtained fractions were chemically profiled by a recently developed dereplication method based on ^13^C nuclear magnetic resonance (NMR) [24]. 

**Figure 4:**
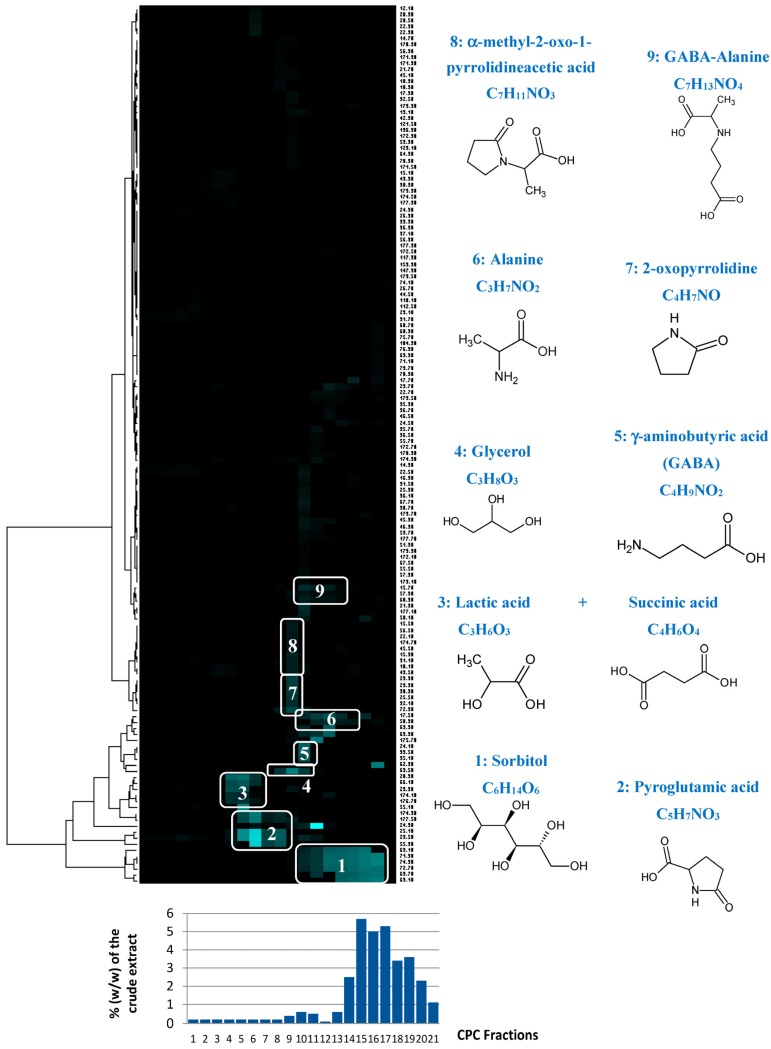
Hierarchical clustering analysis of ^13^C NMR signals detected in the centrifugal partition chromatography (CPC) fractions of the *Rhodosorus marinus* extract, and identification of the major extract constituents.

CPC is a solid-support-free liquid–liquid separation technique involving the distribution and transfer of solutes between at least two immiscible liquid phases according to their distribution coefficient. The absence of a chromatographic solid support avoids irreversible adsorption and/or limits artifact production, ensuring a total recovery of the injected sample. A quantity of 4 g of the crude *Rhodosorus marinus* extract was fractionated in a single run. As this crude extract was obtained by an acid hydrolysis process at elevated temperature, its stability under acidic pH conditions in the presence of acetic acid in the biphasic solvent system was not open to question. The different constituents of the initial extract were eluted in decreasing order of polarity, resulting in 22 successive fractions (denoted F1 to F22) containing simplified mixtures or even pure compounds. The last fraction F22 corresponding to the extrusion step (compounds retained in the stationary phase) did not contain any organic compounds, but represented ≈35% of the injected mass. Since no signal was detected in the ^13^C NMR spectrum of this fraction, we concluded that it was mainly composed of inorganic salts. Fractions from F1 to F21 were directly analyzed by ^13^C NMR for dereplication. Automatic peak picking and alignment of ^13^C signals across spectra of the fraction series resulted in a table with 21 columns (one per fraction) and 144 rows (one per chemical shift bin containing at least one ^13^C NMR signal in at least one fraction). This matrix was submitted to hierarchical clustering analysis (HCA) on the rows. In this way, statistical correlations between ^13^C NMR resonances belonging to a single structure within the fraction series were easily visualized as “chemical shift clusters” in front of the corresponding dendrograms (Figure 4). Several well-defined clusters were intensely colored in blue in the resulting HCA correlation map. Cluster 1 corresponded to an intense cluster of six ^13^C NMR chemical shifts. After entering these chemical shifts into our internal database containing predicted ^13^C NMR data of natural compounds, the structure of sorbitol was proposed as the first hit of over 43 proposals. This structure was successfully confirmed by checking all chemical shifts of sorbitol in raw NMR data of fractions F17, F18, and F19 where the intensity of Cluster 1 was predominant, and by 2D NMR analyses of F18. By applying exactly the same approach, Clusters 2, 4, 5, and 6 were identified as pyroglutamic acid, glycerol, γ-aminobutyric acid, and alanine, respectively. Cluster 3 corresponded to a mixture of lactic acid and succinic acid. Clusters 7, 8, and 9 did not match exactly with database proposals, but they were unambiguously identified as 2-oxopyrrolidine, α-methyl-2-oxo-1-pyrrolidineacetic acid, and GABA-alanine by manually performing the structural elucidation process with the 2D NMR spectra of F13 and F14.

Based on these chemical profiling results, several fractions (F4, F11, F13, F17, F19, and F22) exhibiting significantly different molecular profiles, and containing the main identified *Rhodosorus marinus* metabolites, were selected to be tested again on the in vitro TRPV1 over-expression model, to tentatively determine which metabolite(s) were involved in the neuro-inflammation inhibitory activity observed for the whole extract. 

**Figure 5:**
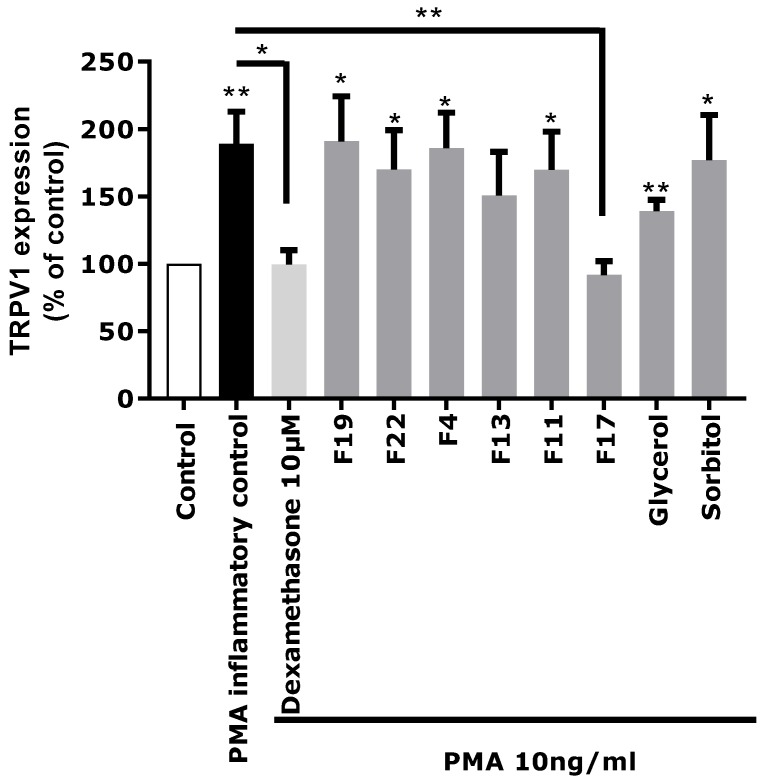
Impact of the CPC fractions on TRPV1 expression in normal human astrocytes (NHA) under PMA-induced inflammatory conditions. Statistical analyses were performed using Student’s *t*-test with *p* < 0.05 *, *p* < 0.01 **.

Each fraction was diluted at the dose estimated in the extract and incubated on astrocytes for 48 h after PMA-induced inflammation. TRPV1 was specifically immunostained and its expression level was analyzed by fluorescence microscopy and quantified by image analysis (Figure 5). As glycerol and sorbitol were detected as major constituents of the extract, their impact on TRPV1 expression was also evaluated using pure reference compounds. As illustrated in Figure 5, the significant over-expression of TRPV1 induced by PMA in astrocytes was reduced by the anti-inflammatory reference dexamethasone. Fractions F4, F11, F13, F19, and F22 showed a similar effect as PMA alone and did not reduce TRPV1 over-expression, indicating that these fractions were not active and unable to modulate the PMA-induced inflammation. Similarly, sorbitol and glycerol tested as pure compounds were not able to reduce the over-expression of TRPV1 induced by PMA. In contrast, fraction F17 induced a significant decrease of TRPV1 expression similar to that observed with the crude *Rhodosorus marinus* extract (see Figure 3). These results suggest that fraction F17 contained one or several metabolites responsible for the biological activity of the *Rhodosorus marinus* extract. 

The NMR chemical profiling of fraction F17 revealed the presence of γ-aminobutyric acid (GABA) and γ-aminobutyric acid alanine (GABA-Ala) as major constituents. These two compounds were thus potential candidates for the soothing effect of the *Rhodosorus marinus* extract. Interestingly, it was recently reported that the subunit of the GABA-B1 receptor (γ-aminobutyric acid receptor B1) can control TRPV1-related sensitization in diverse inflammation settings [25]. Therefore, it can be hypothesized here that GABA, which is an endogenous GABAB1 agonist released from nociceptive nerve endings, exerts an autocrine feedback mechanism limiting TRPV1 sensitization.

**Figure 6:**
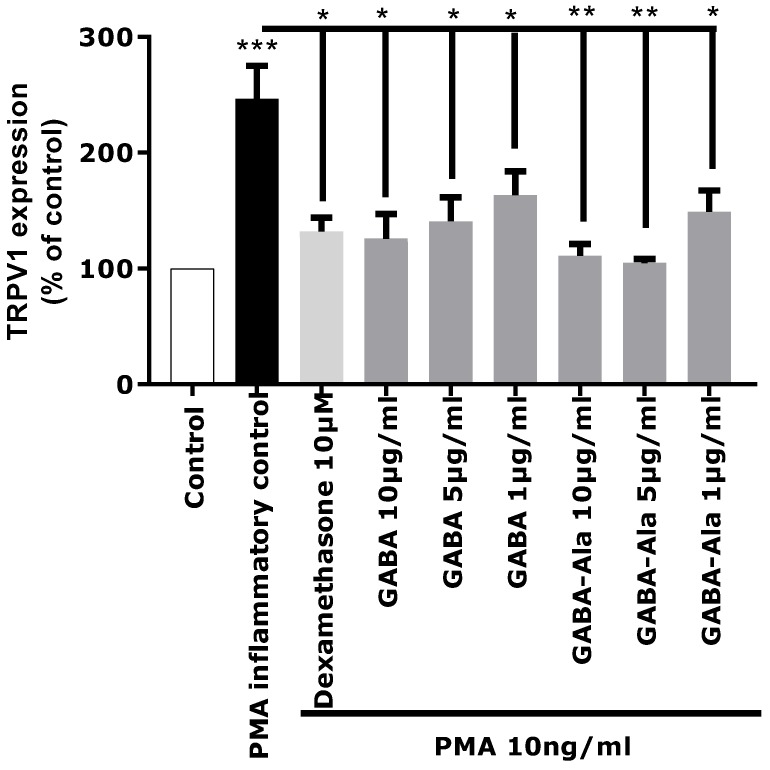
Biological evaluation of pure γ-aminobutyric acid (GABA) and GABA-Alanine on TRPV1 expression in inflammatory conditions. Statistical analyses were performed using Student’s *t*-test with *p* < 0.05 *, *p* < 0.01 **, *p* < 0.001 ***.

The in vitro activity of pure GABA and pure GABA-Ala on neuro-inflammation related to TRPV1 over-expression was therefore evaluated to determine if these two molecules were truly active. GABA and GABA-Ala were tested at the dose defined in the *Rhodosorus marinus* extract. HPLC-quantification of GABA and GABA-Ala in the RDMS extract was evaluated at 220.7 and 277.5 µg/mL, respectively, corresponding to a range of 2.2 to 8.3 µg/mL for a use of Mariliance at 1% or 3%; both compounds were thus tested at 1, 5, and 10 µg/mL. As presented in Figure 6, a significant inhibition of TRPV1 over-expression mediated by PMA was clearly observed for GABA with a dose-dependent effect. These results are in accordance with recently published data describing GABA as a new antagonist of the inflammatory-mediated TRPV1 pain signal through its interaction with the GABA-B1 receptor which reverses the sensitization state [25,26]. Apart from its role in the central nervous system, several works have also reported that GABA can modulate inflammation [27], either by decreasing inflammatory cytokine production [28] or by controlling the cytotoxic immune response and cutaneous hypersensitivity reactions [29,30]. In other contexts, GABA has been shown to play a down-regulating role in the production of pro-inflammatory mediators such as IL-1, IL-6, and matrix metalloproteinase 3 (MMP-3) [31]. 

Regarding GABA-Ala, a significant inhibition of TRPV1 over-expression mediated by PMA was also observed (Figure 6), even with a slightly better effect than that observed with the GABA treatment. To our knowledge, GABA-Ala has never been described previously as an antagonist of the TRPV1 sensitization pathway. GABA-Ala is thus identified here for the first time as an efficient TRPV1 inhibitor. 

## 3. Materials and Methods

### 3.1. Solvents and Reagents

Acetic acid and *n-*butanol were purchased from Carlo Erba Reactifs SDS (Val de Reuil, France). Aqueous solutions were prepared with ultrapure water. Deuterated dimethyl sulfoxide (DMSO-*d*_6_), fluorenylmethyloxycarbonyl (Fmoc) chloride, boric acid, and γ-aminobutyric acid were purchased from Sigma-Aldrich (Saint-Quentin, France).

### 3.2. Rhodosorus marinus Extract

*Rhodosorus marinus* was cultivated at industrial scale at Marine Biotechnology Centre of Excellence of GIVAUDAN (Ile Grande, Pleumeur-Bodou, France). The cells were grown in natural seawater supplemented with Conway medium (Walne, 1966) in ponds of 20 m^3^ under natural light conditions and at a temperature of 23 °C ± 2 °C. The pH was regulated at 7.5 with CO_2_-enriched air. Cultures were grown in a semicontinuous regime and cells were harvested by centrifugation at 17,000 *g*. The extract of *Rhodosorus marinus* was obtained after acid hydrolysis of the biomass followed by neutralization, centrifugation, filtration, and discoloration with activated carbon.

### 3.3. Transcriptomic Analysis on NHEK

NHEK (neonatal, from Lonza CC-2507) were cultivated in Epilife (Invitrogen, Carlsbad, CA, USA, M-EPI-500-A) containing the HKGS (Human Keratinocytes Growth Supplement, Invitrogen S-001-K). The *Rhodosorus marinus* extract was incubated at 3% for 24 h on NHEK. The experiment was performed in triplicate. After 24 h of stimulation, total RNA was extracted using a RNeasy Mini kit (Qiagen, Hilden, Germany, 74106) and its integrity was analyzed by capillary electrophoresis (Agilent RNA 6000Nano kit, Santa Clara, CA, USA, 5067-1511). The difference in terms of genic expression was analyzed by RT-qPCR using a TaqMan card targeting epidermis functions designed by StratiCELL (96 genes). The gene list used in the TaqMan card targeting the epidermis is confidential; consequently, only the significant results are reported here. The analysis was performed using the calculation of ΔΔCt with the B2M (β-2-microglobulin) gene used as a reference housekeeping gene for the data normalization. A gene variation was considered significant if the *p*-value was less than 0.05. 

### 3.4. NGF and IL-1a Quantification by ELISA on Normal Human Epidermal Keratinocytes (NHEK)

Cytotoxicity tests were performed to confirm that the *Rhodosorus marinus* extract was not toxic on NHEK and NHA (data not shown). Pooled normal human epidermal keratinocytes (adult, from Lonza) were seeded in 12-well plates at 100,000 cells per well. After 48 h, cells were washed in PBS (Gibco^TM^, Thermo Fischer Scientific, Illkirch, France) and the culture medium was replaced by KGM (Keratinocytes Growth Medium, Lonza, Basel, Switzerland) without hydrocortisone, an anti-inflammatory molecule that can influence the PMA effect and consequently the secretion of NGF and IL-1a. After 24 h, cells were prestimulated with dexamethasone (10 µM) or *Rhodosorus marinus* extract at 1% and 3% for 2 h at 37 °C. Cells were then stimulated with PMA (phorbol myristate acetate, Sigma) at 10 ng/mL. After 48 h of stimulation at 37 °C, supernatant was collected, the released NGF was quantified by ELISA (Abcam, Cambridge, UK), and the secretion of IL-1a was quantified by ELISA from R&D system (DLA50). For each ELISA, the experimental procedure recommended by suppliers was used and the measurements were performed using a TECAN microplate reader.

### 3.5. IL-1a Quantification by ELISA on RHE Penetration

This part of the work was performed by external service provider StratiCELL (Les Isnes, Belgium). Briefly, NHEK (neonatal, from Lonza, CC-2507) were cultivated in monolayer using Epilife (Invitrogen, M-EPI-500-A) containing the HKGS (Human Keratinocytes Growth Supplement, Invitrogen S-001-K) without hydrocortisone (which has an anti-inflammatory property). Cells were maintained in a humid atmosphere at 37 °C with 5% CO_2_. The formulas presented in Table 1 were topically applied for 2 h on reconstructed human epithelia (RHE/001, lot VB1014/7 from StratiCELL) in contact with the keratinocyte monolayer through a co-culture system using an insert over the whole treatment period.

RHE were previously obtained by air–liquid interface culture for 14 days in adequate culture media in a humid atmosphere at 37 °C with 5% CO_2_. After 2 h of prestimulation with dexamethasone or *Rhodosorus marinus* extract formulated in cream, the inflammatory condition was induced by PMA (10 ng/mL, Sigma), associated with calcium ionophore (2 µM, Sigma A23187) added in the culture media, in contact with the keratinocyte monolayer (bottom compartment) for 24 h and 48 h. The supernatant in contact with the keratinocyte monolayers was then collected and IL-1a release was quantified by ELISA (R&D system, DLA50) according to the experimental procedure recommended by suppliers and measured using a TECAN microplate reader.

### 3.6. TRPV1 Expression by Fluorescent Immunostaining

Normal human astrocytes (NHA, Lonza) were seeded at 60,000 cells per well in 24-well plates containing glass slides precoated with poly-l-lysine (Gibco) for 30 min at 37 °C. After 48 h, cells were pretreated for 2 h at 37 °C with dexamethasone (Sigma) at 10 µM, an anti-inflammatory molecule, or with the *Rhodosorus marinus* extract at 1% and 3% in the presence of 2 µM Ica (calcium ionophore). Cells were then stimulated with PMA (phorbol myristate acetate, 10 ng/mL) at 37°C to induce in vitro inflammation. After 24 h, cells were permeabilized for 5 min at RT with buffer containing 2% paraformaldehyde (PFA) and 0.5% Triton X100 (Sigma) in PBS. After three washes in filtered PBS, cells were fixed in 2% PFA in PBS for 10 min at RT. After three washes in PBS, specific sites were saturated with a 3% BSA (Sigma) solution containing 0.1% Tween20 (Sigma) in PBS for 1 h at RT. TRPV1 receptor was stained with a specific anti-TRPV1 primary antibody (Abcam ab74855) diluted at 1/500 in saturation buffer diluted at 1/10 (0.3% BSA solution containing 0.01% Tween 20) and incubated overnight at 4 °C in a humid chamber. After three washes in PBS containing 0.3% Triton X100, cells were incubated with secondary antibody coupled to Alexafluor488 diluted at 1/500 supplemented with Hoechst (10 µg/mL) and incubated 2 h at RT protected from light. The cells were washed as previously and mounted on slides with a specific mounting medium; staining was analyzed by classical fluorescence microscopy (Zeiss, Kwai Chung, Hong Kong) and quantified by Image J.

The same experiment was also performed on pure GABA (γ-aminobutyric acid) or GABA-Alanine at the following concentrations (in the presence of 2 µM ICa): dexamethasone 10 µM, GABA or GABA-Ala 10 µg/mL, GABA or GABA-Ala 5 µg/mL, and GABA or GABA-Ala 1 µg/mL.

### 3.7. Centrifugal Partition Chromatography

The fractionation process was developed on a lab-scale CPC column of 303.5 mL capacity (FCPE300^®^, Rousselet Robatel Kromaton, Annonay, France) containing seven circular partition disks and engraved with a total of 231 oval partition twin-cells (≈1 mL per twin cell). The liquid phases were pumped by a KNAUER Preparative 1800 V7115 pump (Berlin, Germany). Fractions were collected by a Pharmacia Superfrac collector (Uppsala, Sweden). A two-phase solvent system composed of *n-*butanol, acetic acid, and water (4/1/5, *v*/*v*/*v*) was thoroughly equilibrated in a separatory funnel. After decantation, the upper and lower phases were separated. The crude dry extract (4 g) of *Rhodosorus marinus* was dissolved in a mixture of upper/lower phases (1:1, *v*/*v*) to ensure that the liquid phases were in equilibrium in the sample solution. The aqueous phase was used as stationary phase and maintained inside the column by application of a constant centrifugal force field (1200 rpm). The sample solution was loaded into the column through a 35 mL sample loop. After sample injection, the flow rate was progressively increased from 0 to 20 mL/min over 3 min and then maintained at 20 mL/min over the whole experiment. The organic mobile phase was pumped for 60 min in the ascending mode. Then, the aqueous stationary phase was extruded by pumping the organic mobile phase in the descending mode under 1200 rpm in order to recover the most hydrophilic substances retained inside the column. In total, 21 fractions of 60 mL were obtained during elution (F1 to F21) and 1 fraction of 150 mL was recovered from the extrusion step (F22). 

### 3.8. Chemical Profiling of Rhodosorus marinus Metabolites

An NMR-based dereplication method was used to identify the major metabolites contained in the *Rhodosorus marinus* extract. For this purpose, all fractions from F1 to F22 were dried under vacuum at 50 °C and a maximum of 20 mg of each was dissolved in 600 µL of DMSO-*d*_6_. NMR analyses were performed at 298 K on a Bruker Avance AVIII-600 spectrometer (Karlsruhe, Germany) equipped with a cryoprobe optimized for ^1^H detection and with cooled ^1^H, ^13^C, and ^2^H coils and preamplifiers. ^13^C NMR spectra were acquired at 150.91 MHz. A standard zgpg pulse sequence was used with a 90° pulse, an acquisition time of 0.909 s, and a relaxation delay of 3 s. For each NMR sample, 1024 scans were co-added to obtain a satisfactory signal-to-noise ratio. The spectral width was 238.9070 ppm and the receiver gain was set to the highest possible value. A 1 Hz line broadening filter was applied to each FID prior to Fourier transformation. The spectra were manually phased and baseline corrected using the TOPSPIN 3.2 software (Bruker, Kowloon, Hong Kong) and calibrated on the central resonance (δ 39.80 ppm) of DMSO-*d*_6_. A minimum intensity threshold of 0.3% (relative to the most intense signal of each spectrum) was then used to automatically collect all positive ^13^C NMR signals while avoiding potential noise artifacts. Each peak list was then converted into a text file. The collected peaks in the fraction series were aligned by using an in-house algorithm written in the Python language. The principle was to divide the ^13^C spectral width (from 0 to 200 ppm) into regular bins, i.e., chemical shift intervals of ∆δ = 0.2 ppm, and to associate the absolute intensity of each ^13^C NMR peak with the corresponding bin. The resulting table was imported into the PermutMatrix version 1.9.3 software (LIRMM, Montpellier, France) for clustering analysis on raw peak intensity values. The classification was performed on the rows only, i.e., on the chemical shift bins. The Euclidean distance was used to measure the proximity between samples and Ward’s method was performed to agglomerate the data. The resulting ^13^C chemical shift clusters, corresponding to the major carbon skeletons present in the crude *Rhodosorus marinus* extract, were visualized as dendrograms on a two-dimensional map (Figure 4). The higher the intensity of ^13^C NMR peaks, the brighter the color on the map. For metabolite identification, each ^13^C NMR chemical shift cluster obtained from HCA was submitted to a locally built database (ACD/NMR Workbook Suite 2012 software, ACD/Labs, Toronto, ON, Canada) containing the structures and predicted ^13^C NMR chemical shifts of natural metabolites (*n* = 2800 in December 2017). A ^13^C NMR chemical shift tolerance of ±2 ppm was used for the NMR database search.

Additional 2D NMR experiments were performed on the same Bruker Avance AVIII-600 spectrometer to confirm the structures of the proposed metabolites. HSQC, HMBC, and COSY spectra were recorded using standard Bruker pulse programs. For the acquisition of HSQC spectra (pulse sequence hsqcedetgpsisp2.2), the size of FID was set at 1024 in the F2 dimension and 512 in the F1 dimension, and the number of scans was set at 4. For the acquisition of HMBC spectra (pulse sequence hmbcetgpl3nd), the size of FID was set at 2048 in the F2 dimension and 512 in the F1 dimension, and the number of scans was 4. For the acquisition of COSY spectra (pulse sequence cosygpqf), the size of FID was set at 4096 in the F2 dimension and 512 in the F1 dimension, and the number of scans was set at 4.

### 3.9. Biological Evaluation of the CPC Fractions

The CPC fractions F4, F11, F13, F17, F19, and F22 containing the major characteristic compounds identified in the *Rhodosorus marinus* extract were selected to be tested for their impact on TRPV1 expression in our model up-regulated by PMA. Human astrocytes (NHA) were seeded at 60,000 cells per well in 24-well plates containing glass slices already coated with poly-l-Lysine (30 min at 37 °C). After 48 h, cells were stimulated for 2 h at 37 °C with dexamethasone (Sigma) or with the CPC fractions of the *Rhodosorus marinus* extract at the following concentrations: dexamethasone 10 µM, F19 50 µg/mL, F22 50 µg/mL, F13 10 µg/mL, F4 5 µg/mL, F11 5 µg/mL, and F17 50 µg/mL. These concentrations were selected on the basis of active metabolite concentration estimation as revealed by NMR analyses. Dexamethasone and the tested fractions were all mixed with 2 µM of calcium ionophore (Ica 2 µM) in order to better induce the inflammation process. As sorbitol and glycerol were two molecules largely present in the *Rhodosorus marinus* extract, commercial Sorbitol at 50 µg/mL (Sigma) and Glycerol at 10 µg/mL (Sigma) were also tested in order to verify if these molecules could have an impact on TRPV1 expression in our experimental conditions. After 2 h of prestimulation with the different fractions or dexamethasone, cells were then stimulated with PMA (10 ng/mL, Sigma) for 24 h at 37 °C to induce in vitro inflammation. After 24 h of stimulation, the immunocytochemistry test was performed as described in Section 3.6.

### 3.10. Synthesis of GABA-Alanine

In a 500 mL round-bottomed flask, 4-aminobutanoic acid (10 g, 97 mmol) was dissolved in a sodium hydroxide solution (7.8 g, 195 mmol) in water (20 mL). The resulting colorless solution was cooled with an ice bath down to 12 °C. 2-bromopropanoic acid (15 g, 98 mmol) was added dropwise at temperature below 25 °C. Additional NaOH (2 g) in 6 g water was added and stirring was continued for 2 h at temperature between 30 and 40 °C. After removal of water under reduced pressure at 40 °C, the remaining residue was added to 100 mL of methanol. The insoluble sodium bromide was removed by filtration. Diethyl ether (300 mL) was added to the methanol solution until complete precipitation occurred. The white precipitate was washed with ether and dried under vacuum at 30 °C. A quantity of 15 g of sodium 4-((1-carboxylatoethyl)amino)butanoate (intermediate) was yielded with a purity of 95% as revealed by NMR analysis.

**Figure 7:**
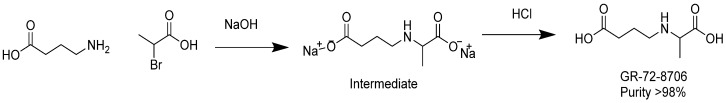
Synthesis of GABA-Alanine.

In a 250 mL round-bottomed flask, the intermediate sodium 4-((1-carboxylatoethyl)amino)butanoate (6.8 g) was dissolved in 20 mL water. The solution was cooled to 4 °C with an ice bath and the pH of the mixture was adjusted to pH 6–7 with diluted aq. HCl. The solution was diluted with acetone until precipitation occurred and the mixture was then placed at 4 °C overnight. The next day, the white solid was filtered, washed with acetone, and dried in a vacuum oven to obtain 4.5 g of product at purity ca. 95% as revealed by NMR. The product was dissolved in 30 mL of DMSO and agitated for 15 min. The insoluble solid was filtered, and washed successively with methanol, acetone, and ether. The white solid was dried in an oven under vacuum at 50 °C. As a result, 2.5 g of GABA-Alanine was yielded with purity greater than 98% as revealed by NMR. The chemical synthesis of GABA-Ala is described in Figure 7.

### 3.11. HPLC Quantification of GABA and GABA-Alanine

The *Rhodosorus marinus* extract and GABA and GABA-Ala standards were diluted in HCl 0.01 M. Stock solutions of standards were prepared at 1 mg/mL. Standard curves were realized by mixing GABA and GABA-Ala at concentrations ranging from 18.75 to 300 µg/mL. The derivatization procedure was performed by mixing 300 µL of the amino acids solution or RDMS extract, 600 µL of borate buffer 200 mM pH 10, and 600 µL of Fmoc 15 mM. After 5 min vortexing, samples were filtered through 0.45 µm nylon. HPLC analyses were performed on an Agilent 1100 HPLC system equipped with a UV detector set at 263 nm. Samples were injected with a 10 µL loop onto an Eclipse XDB 5 µm C18 column (250 × 4.6 mm) at room temperature. The flow rate was set at 1 mL/min using 50 mM acetate buffer (pH 4.2 with NaOH) as eluent A and acetonitrile as eluent B. Amino acids were separated using the following linear gradient (min/%B): 0/35, 15/35, 30/65, 31/100, 36/100, 37/35, 42/35.

## 4. Conclusions

In conclusion, we demonstrated that the red microalgae *Rhodosorus marinus* contains several polar metabolites that are able to control the release of pro-inflammatory cytokines (IL-1α) and neuro-inflammation mediators, and to positively regulate skin sensitization mechanisms related to the TRPV1 receptors. The NMR chemical profiling and bioactivity-guided fractionation of the extract revealed the presence of two active molecules, namely, γ-aminobutyric acid (GABA) and its structural derivative GABA-alanine, with the capacity to significantly decrease TRPV1 over-expression in normal human astrocytes under PMA-induced inflammatory conditions. This *Rhodosorus marinus* extract therefore provides interesting perspectives for the treatment of sensitive skin, atopia, dermatitis, or psoriasis.

## Figures and Tables

**Table 1:** Tested formulas for IL-1a quantification on reconstructed human epithelia. **Q**S: Quantum Satis.

Ingredient	Control Cream	Test Cream
Xyliance	4%	4%
Kendi oil	0.5%	0.5%
Phenoxyethanol	0.5%	0.5%
Aqua	QS	QS
*Rhodosorus marinus* extract/Dexamethasone		1% or 3%/0.2%
